# Supplementary material for: Serum amyloid P component is an essential element of resistance against Aspergillus fumigatus
Source: Nat Commun. 2021 Jun 18;12:3739. doi: 10.1038/s41467-021-24021-y (PMC8213769; doi:10.1038/s41467-021-24021-y)
Supplement: Supplementary file 1 — Supplementary Information [file 41467_2021_24021_MOESM1_ESM.pdf]

SUPPLEMENTARY INFORMATION

Serum Amyloid P component is an essential element of resistance against *Aspergillus fumigatus*

Andrea Doni[1], Raffaella Parente[1], Ilaria Laface[1,†], Elena Magrini[1], Cristina Cunha[2, 3], Federico Simone Colombo[1,] João F. Lacerda[4, 5], António Campos Jr.[6], Sarah N. Mapelli[1], Francesca Petroni[1], Rémi Porte[1], Tilo Schorn[1], Antonio Inforzato[1, 7], Toine Mercier[8, 9], Katrien Lagrou[9, 10], Johan Maertens[8, 9], John D. Lambris[11], Barbara Bottazzi[1], Cecilia Garlanda[1, 7], Marina Botto[12], Agostinho Carvalho[2, 3], Alberto Mantovani[1, 7, 13, *]

[1], IRCCS Humanitas Clinical and Research Center, Rozzano, Milan, Italy

[2], Life and Health Sciences Research Institute (ICVS), School of Medicine, University of Minho, Braga, Portugal

[3], ICVS/3B's - PT Government Associate Laboratory, Guimarães/Braga, Portugal

[4], Instituto de Medicina Molecular, Faculdade de Medicina de Lisboa, Lisboa, Portugal

[5], Serviço de Hematologia e Transplantação de Medula, Hospital de Santa Maria, Lisboa, Portugal

[6], Serviço de Transplantação de Medula Óssea (STMO), Instituto Português de Oncologia do Porto, Porto, Portugal

[7], Humanitas University of Milan, Pieve Emanuele, Milan, Italy

[8], Department of Hematology, University Hospitals Leuven, Leuven, Belgium

[9], Department of Microbiology, Immunology and Transplantation, KU Leuven, Leuven, Belgium

[10], Department of Laboratory Medicine and National Reference Centre for Mycosis, University Hospitals Leuven, Leuven, Belgium

[11], Department of Pathology and Laboratory Medicine, Perelman School of Medicine, University of Pennsylvania, Philadelphia, Pennsylvania, PA 19104, USA

[12], Imperial College London, London, UK

[13], The William Harvey Research Institute, Queen Mary University of London, London, UK

[†], current affiliation: Department of Translational Medicine and for Romagna, University of Ferrara, Ferrara, Milan, Italy

Supplementary Table 1. **Susceptibility of *Apcs*$^{-/-}$ mice to pulmonary aspergillosis**.

	MST (day)	Dead total (n)	Survival (%)	*P* value
wt	nd	7/39	82.0	
Apcs$^{-/-}$	3	34/46	26.1	<0.0001

Summary of the survival experiments performed in *Apcs*$^{-/-}$ mice i.t. injected with $5x10^7$ *A. fumigatus* conidia. MST, median survival time. *P*, Fisher's Exact test (two-sided).

Supplementary Table 2. **Baseline characteristics of transplant recipients enrolled in the study**.

Variables	IPA (n=111)	No IPA (n=372)	*P* value
Age at transplantation, no (%)			
≤20 years	16 (14.4)	81 (21.8)	0.150
21 – 40 years	30 (27.0)	108 (29.0)	
>40 years	65 (58.6)	183 (49.2)	
Gender, no (%)			
Female	48 (43.2)	158 (42.5)	0.870
Male	63 (56.8)	214 (57.5)	
Underlying disease, no. (%)			
Acute leukemia	61 (55.0)	197 (53.0)	0.223
Chronic lymphoproliferative diseases	16 (14.4)	67 (18.0)	
Myelodysplastic/myeloproliferative diseases	17 (15.3)	34 (9.1)	
Chronic myeloproliferative diseases	8 (7.2)	21 (5.6)	
Aplastic anemia	6 (5.4)	29 (7.8)	
Other	3 (2.7)	24 (6.5)	
Transplantation type, no. (%)			
Matched, related	36 (32.4)	175 (47.0)	0.009
Matched, unrelated	40 (36.0)	91 (24.5)	
Mismatched, related	0 (0.0)	8 (2.2)	
Mismatched, unrelated	35 (31.5)	98 (26.3)	
Graft source, no. (%)			
Peripheral blood	91 (82.0)	306 (82.3)	0.645
Bone-marrow	19 (17.1)	57 (15.3)	

Cord blood	1 (0.9)	9 (2.4)	
Disease stage, no. (%)			
First complete remission	59 (53.2)	204 (54.8)	0.940
Second or subsequent remission, or relapse	19 (17.1)	63 (17.0)	
Active disease	33 (29.7)	105 (28.2)	
Conditioning regimen, no (%)			
RIC	79 (71.2)	250 (67.2)	0.452
Myeloablative	32 (28.8)	122 (32.8)	
CMV serostatus of donor and recipient, no. (%)			
D-/R+ or D+/R+	94 (84.7)	331 (89.0)	0.214
D-/R- or D+/R-	17 (15.3)	41 (11.0)	
Duration of neutropenia, mean days (range)†	13.2 (8 – 39)	14.0 (5 – 35)	0.504
Acute GVHD, no. (%)			
No GVHD or grades I – II	77 (69.4)	325 (87.4)	<0.001
Grades III – IV	34 (30.6)	47 (12.6)	
Antifungal prophylaxis, no. (%)‡			
Fluconazole	55 (49.6)	146 (39.2)	0.036
Posaconazole	31 (27.9)	118 (31.7)	
Other	9 (8.1)	15 (4.0)	
None or unknown	16 (14.4)	87 (23.4)	

Chronic lymphoproliferative diseases included cases of chronic lymphocytic leukemia, multiple myeloma, and B- and T-cell lymphomas. Chronic myeloproliferative diseases included cases of chronic myelogenous leukemia and primary myelofibrosis. Other diseases included cases of

idiopathic medullar aplasia, lymphohistiocytosis, hemoglobinopathies and paroxysmal nocturnal hemoglobinuria. RIC, reduced intensity conditioning; CMV, cytomegalovirus; D, donor; R, recipient; GVHD, graft-versus-host-disease. †Neutropenia was defined as $\leq 0.5\times10^9$ cells/L. ‡Other antifungals used in prophylaxis included voriconazole, liposomal amphotericin B, itraconazole and caspofungin. *P* values were calculated by Fisher's exact probability t-test or Student's t-test (two-sided) for continuous variables. Variables with $P<0.15$ were included in the multivariate model.

Supplementary Table 3. **Sequences of primers used in the genotyping study**.

Assays ID	FAM Allele	HEX Allele	Sequence
APCS rs2808661	A	G	ATCAATGGGACACCTTTGGTGAAAAAGGGTCTGCGACAGGGTTACTTTGT[A/G]GAAG CTCAGCCCAAGATTGTCCTGGGGCAGGAACAGGATTCCTATGGGGG
APCS rs3753869	A	C	TTCTCTCCTCTTCTTTGAGTCCTTGGTCATTGAGTGTATATCTAATCCAT[A/C]TTTGGT GATGACCCCACTAATACATTTATTCACTAGCTCTCAGTTACTAC

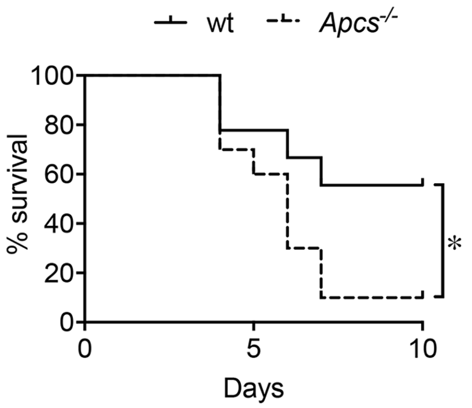

Supplementary Figure 1. **Susceptibility of *Apcs$^{-/-}$* mice to *A. flavus***. survival of wt and *Apcs$^{-/-}$* mice after i.t. injection of $5x10^7$ conidia; wt, n=9; *Apcs$^{-/-}$*, n=10. An independent experiment shown. *, *P*=0.05 (Log-rank Mantel-Cox test).

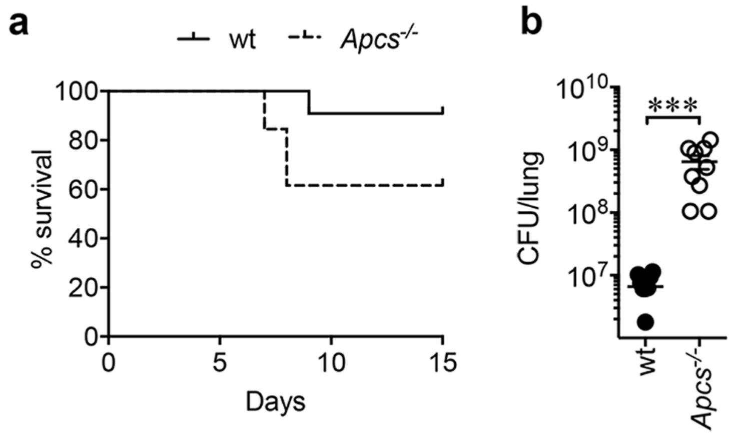

Supplementary Figure 2. **Susceptibility of *Apcs*$^{-/-}$ mice to *A. terreus*.** **a**, survival of wt and *Apcs*$^{-/-}$ mice after i.t. injection of 1x10^{8} conidia; wt, n=11; *Apcs*$^{-/-}$, n=13. **b**, number of CFU per lung at 16h after infection with 1x10^{8} conidia. Each spot corresponds to a single mouse; wt, n=8; *Apcs*$^{-/-}$, n=9. One independent experiment performed. Mean±SEM. ***, *P*=0.002 (two-sided, unpaired t-test).

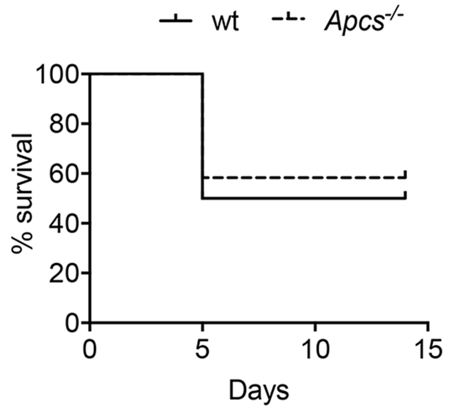

Supplementary Figure 3. **No relevance of SAP-deficiency in resistance to *Candida albicans* infection**. *in vivo* model of *C. albicans* dissemination in wt (n=10) and *Apcs*$^{-/-}$ (n=12) mice. Monitoring of the survival after i.v. injection of *C. albicans* blastospores (5x10^6). An independent experiment shown.

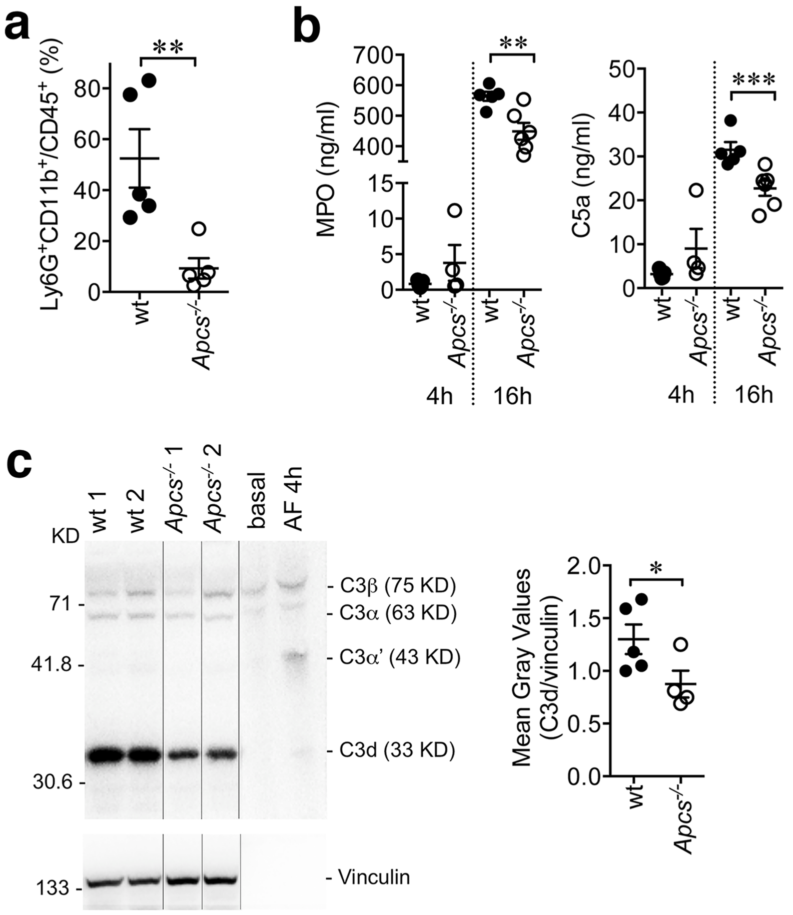

Supplementary Figure 4. **Impaired neutrophil recruitment in lungs of *Apcs$^{-/-}$* mice in response to *A. fumigatus*.** **a**, FACS analysis of neutrophil recruitment in lung at 16h. N=5 wt and n=5 *Apcs$^{-/-}$* mice. Mean±SEM. **, *P*=0.008 (two-sided, Mann-Whitney *U* test). **b**, MPO and C5a levels in BALFs of wt (4h, n=5; 16h, n=5) and *Apcs$^{-/-}$* (4h, n=4; 16h, n=6) mice after injection of 5x10^{7} AF conidia (16h). MPO, **, *P*=0.008; C5a, ***, *P*=0.004 (two-sided, Mann-Whitney *U* test). **a**, **b**, One independent experiment shown out of two performed. Mean±SEM. **c**, left, Western blot analysis of complement C3 fragments in lung lysates 4h after injection of 5x10^{7} AF conidia. N=5 wt and n=4 *Apcs$^{-/-}$* mice, two representative loading per genotype are shown (10μg/lane of proteins); 1μl/lane of mouse plasma in basal conditions and 4h after AF injection. Vinculin used as loading control is also shown. Right, results are expressed as mean±SEM grey values of C3d/vinculin. One independent experiment shown. *, *P*=0.03 (two-sided, unpaired t-test).

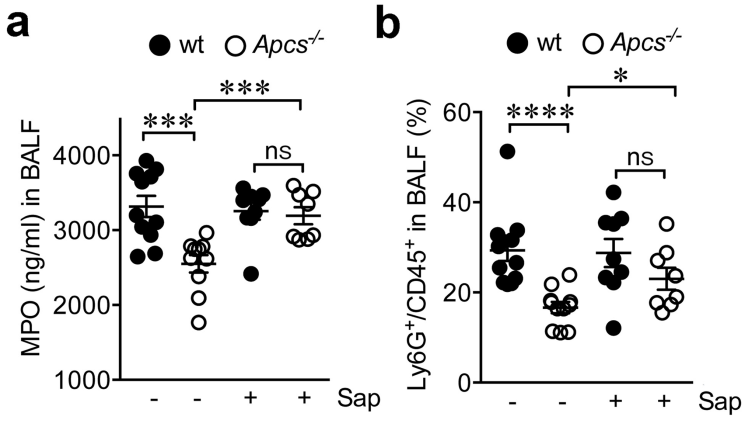

Supplementary Figure 5. **Murine SAP-opsonization rescues the defective neutrophil recruitment in lungs of *Apcs*$^{-/-}$ mice**. **a**, levels of MPO in BALFs of mice (16h). N=11, wt; n=10, *Apcs*$^{-/-}$; n=9, wt + Sap; n=8, *Apcs*$^{-/-}$ + Sap. Mean±SEM. Wt vs. *Apcs*$^{-/-}$, ***, *P*=0.0006; *Apcs*$^{-/-}$ vs. *Apcs*$^{-/-}$ + Sap, ***, *P*=0.001 (two-sided, unpaired t-test). **b**, FACS analysis of neutrophil recruitment in lung (16h). N=12, wt; n=11, *Apcs*$^{-/-}$; n=9, wt + Sap; n=8, *Apcs*$^{-/-}$ + Sap. Mean±SEM. *, *P*=0.02 (two-sided, unpaired t-test); ****, *P*<0.0001 (two-sided, Mann-Whitney *U* test). **a**, **b**, an independent experiment is shown.

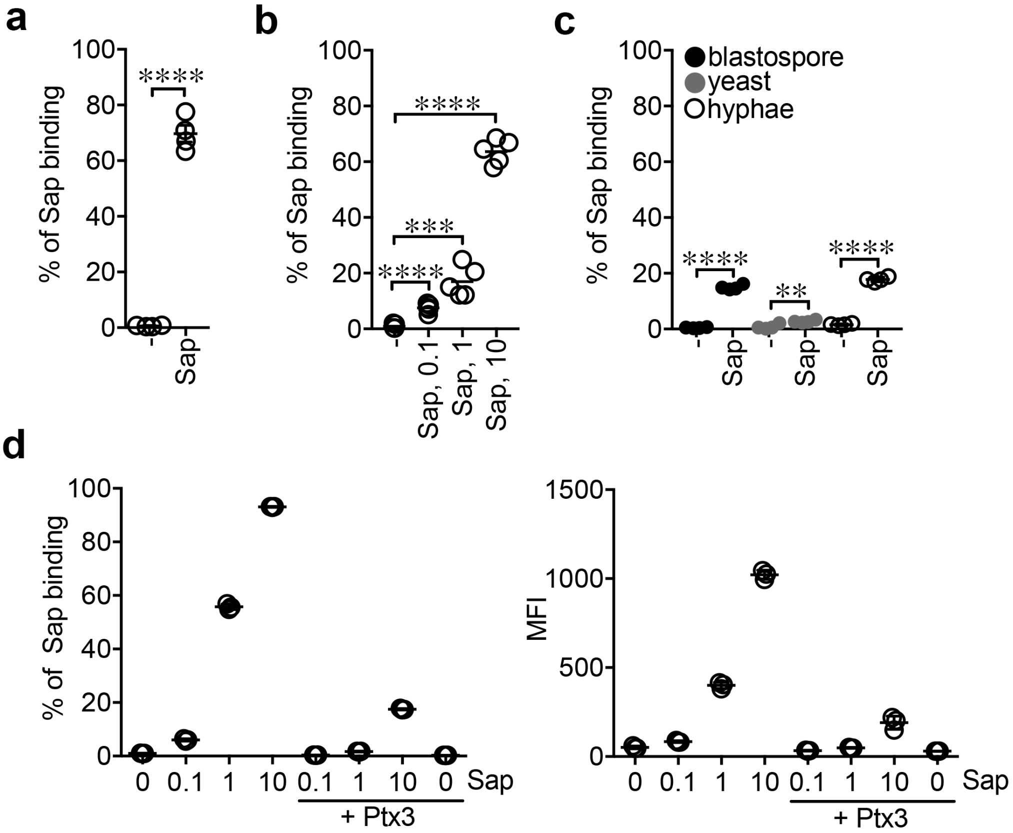

Supplementary Figure 6. **SAP binding to *Aspergillus* and competition with PTX3**. **a**, **b**, **c**, FACS analysis of the binding of murine b-SAP (10µg/ml, **a**, **c**; 0.1-10µg/ml, **b**) to viable *A. flavus* conidia of AF (1x10^8) (**a**), *A. terreus* conidia (1x10^8) (**b**) and blastospore, yeast and hyphae of *C. albicans* (1x10^8) (**c**). **a**, **b**, **c**, one quadruplicate experiment performed. Mean±SD. **a**, ****, *P*<0.0001 (two-sided, unpaired t-test). **b**, ***, *P*=0.0002, ****, *P*<0.0001 (two-sided, unpaired t-test). **c**, ****, *P*<0.0001, **, *P*=0.009 (two-sided, unpaired t-test). **d**, FACS analysis of murine SAP (range from 0.1 to 10µg/ml) binding to viable AF conidia (1x10^8) in presence of murine Ptx3 (50µg/ml). An anti-Sap monoclonal antibody (non-reactive to Ptx3) was used. Mean±SD of a triplicate. **a**, **b**, **c**, **d**, independent experiments are shown.

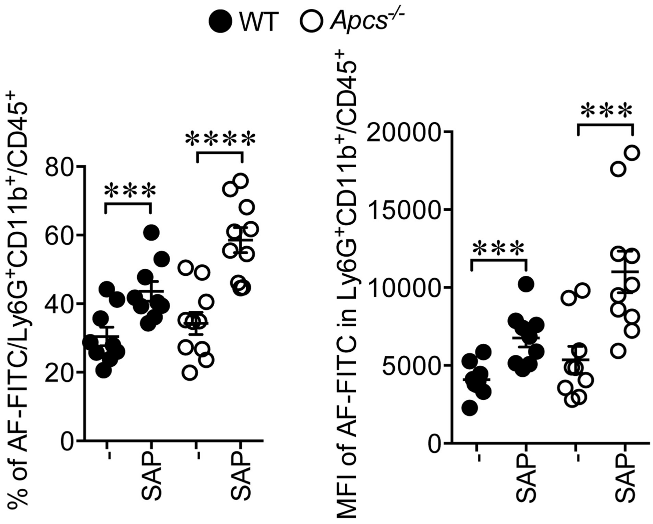

Supplementary Figure 7. **Effect of human SAP on phagocytic activity by murine neutrophils**. FACS analysis of neutrophil phagocytosis in blood of wt (n=9) and *Apcs*$^{-/-}$ (n=10) mice after exposure with FITC-labelled AF conidia (5x10^{6}/200µl of blood) opsonized or not with human SAP. Figure refers to 2 experiments performed and merged. Mean±SEM. Left, ***, *P*=0.004; ****, *P*<0.0001. Right, wt, ***, *P*=0.001; *Apcs*$^{-/-}$, ***, *P*=0.004 (two-sided, unpaired t-test).

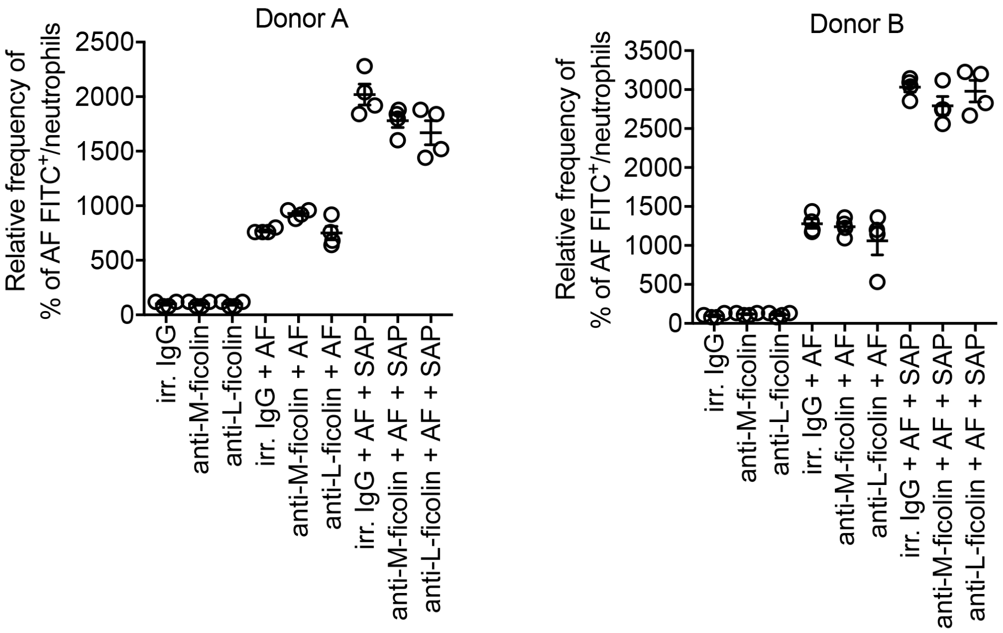

Supplementary Figure 8. **No effect from the use of antibodies specific for ficolins on SAP-mediated phagocytosis by human neutrophils *in vitro***. FACS analysis of phagocytosis (30 min) by freshly isolated human neutrophils (1x10^5). FITC-labelled AF conidia (1x10^6) were pre-opsonized or not with human SAP (2x10^8 conidia and 100µg SAP/ml). Phagocytosis was performed in the presence of 10% autologous serum and of mAbs (10µg/ml) anti-M- and L-ficolin or an irrelevant mouse IgG_1. Each spot corresponds to a replicate of one experiment performed in quadruplicate. Mean±SEM.

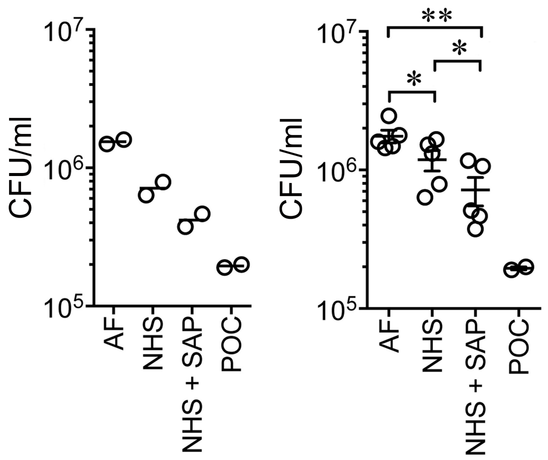

Supplementary Figure 9. **SAP-mediated killing of *A. fumigatus* conidia**. Assessment as CFU count per ml of the AF viability performed in normal human serum (30%) with or without human SAP opsonisation. POC, Posaconazole. Two independent experiments performed in duplicate or quadruplicate are shown. Left, mean; right, Mean±SEM. **, *P*=0.008; *, *P*=0.05, AF vs. NHS; *, *P*=0.04, NHS vs. NHS + SAP (two-sided, unpaired t-test).

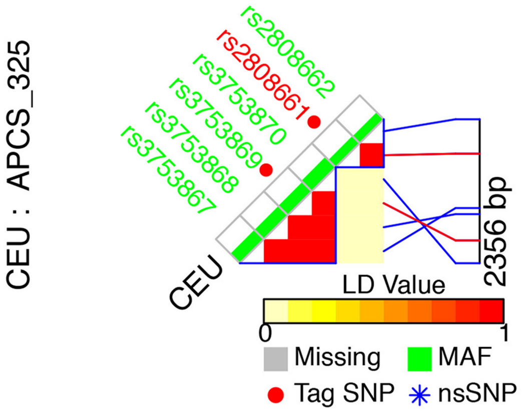

Supplementary Figure 10. **Graphical view of the *Apcs* SNPs selected for genotyping study**. SNPs were selected using the publically available sequencing data from the Pilot 1 of the 1000 Genomes Project for the CEU population.

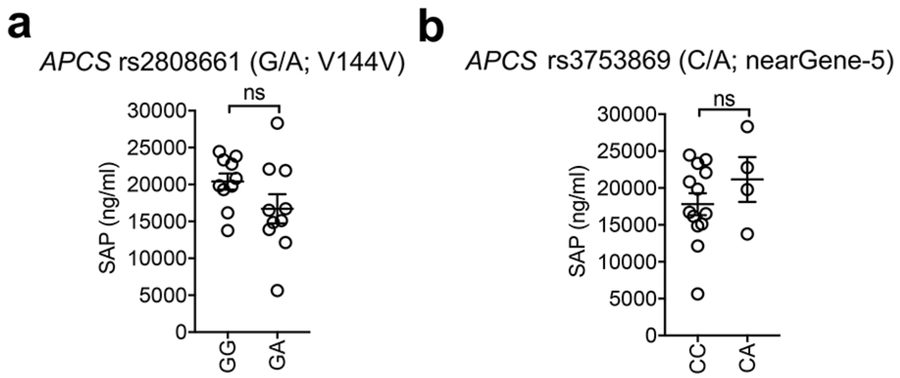

Supplementary Figure 11. **SAP levels in genotypes GG, GA and CC, CA in recipient rs2808661 and rs3753869 SNPs**. **a**, **b**, measurement of SAP levels in recipent haplotypes GG vs. GA or CC vs. CA, respectively for SNPs rs2808661 (GG, n=10; GA, n=10) (**a**) and rs3753869 (**b**) (CC, n=13; CA, n=4). **a**, **b**, Mean±SEM. **a**, two-sided, unpaired t-test. **b**, two-sided, Mann-Whitney *U* test.

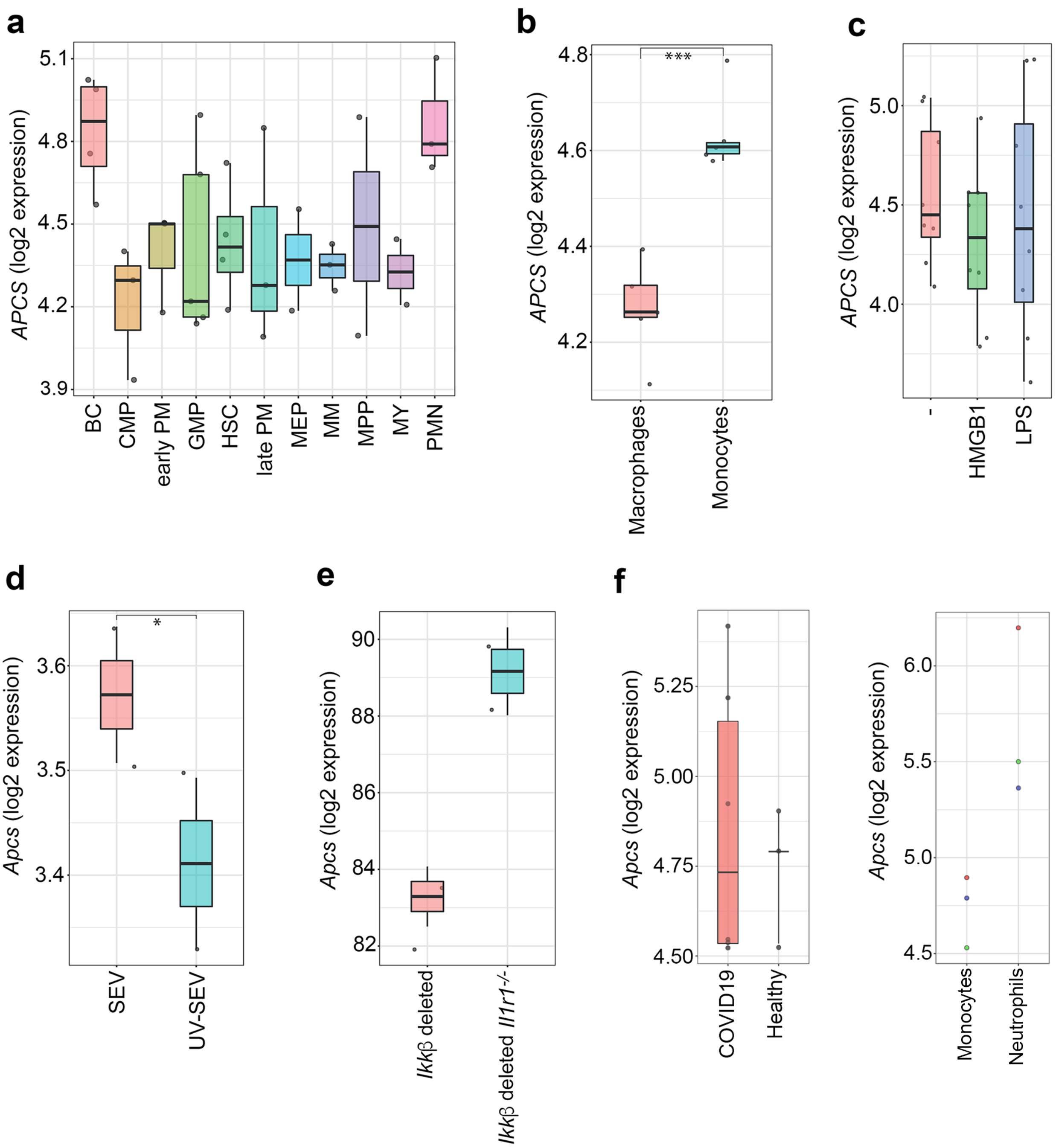

Supplementary Figure 12. ***In silico* analysis of *Apcs* expression in myeloid cells**. Expression data obtained in: **a**, BM cells enriched for CD34$^+$ hematopoietic stem and progenitor cells from healthy donors from the GEO data series GSE42519. BC, band cell; CMP, common myeloid progenitor cell; early PM, early promyelocyte; GMP, granulocyte monocyte progenitor; HSC, hematopoietic stem cell; late PM, late promyelocyte; MEP, megakaryocyte-erythroid progenitor cell; MM, metamyelocyte; MPP, multipotential progenitor; MY, myelocyte; PMN, polymorphonuclear cell; **b**,

macrophages and monocytes of patients with symptoms of acute coronary syndrome and undergone to coronary angiography from the GEO data series GSE11430; **c**, peripheral blood neutrophils isolated from septic patients and treated *in vitro* with LPS or HMGB1 derived from the GEO data series GSE3037; **d**, murine macrophage isolated from wt mice and inoculated with Sendai virus (SeV) or UV-inactivated SeV (UV-SeV) from the GEO data series GSE2935; **e**, murine neutrophils derived from wt and *Il-1r1*$^{-/-}$-deficient mice after treatment with Ikkβ inhibitors obtained from the GEO series GSE25211; **f**, left, *APCS* expression derived from bulk RNA-Seq of human peripheral monocytes of COVID-19 patients and healthy donors. **f**, right, *APCS* expression derived from bulk RNA-Seq of human peripheral monocytes and neutrophils from three healthy individuals. Matched donors are indicated with the same colour code. Data are retrieved from the GEO series GSE160351 and GSE163533. **a**-**f**, Boxplots are represented following ggplot2 criteria: lower and upper box borders represent 25th and 75th percentile, respectively. Median is reported within IQR. Extremities of vertical lines indicate respectively the computed minima and maxima derived as 25th percentile -1.5 * IQR and 75th percentile +1.5 * IQR. Samples below and above these limits are considered potential outliers. **b**, **d**, Differential expressions were tested with the eBayes algorithm implemented in Limma, based on the "empirical Bayes moderation of the variance estimates" assuming a two-sided hypothesis. **b**, ***, *P*=0.002; **d**, *, *P*=0.02.

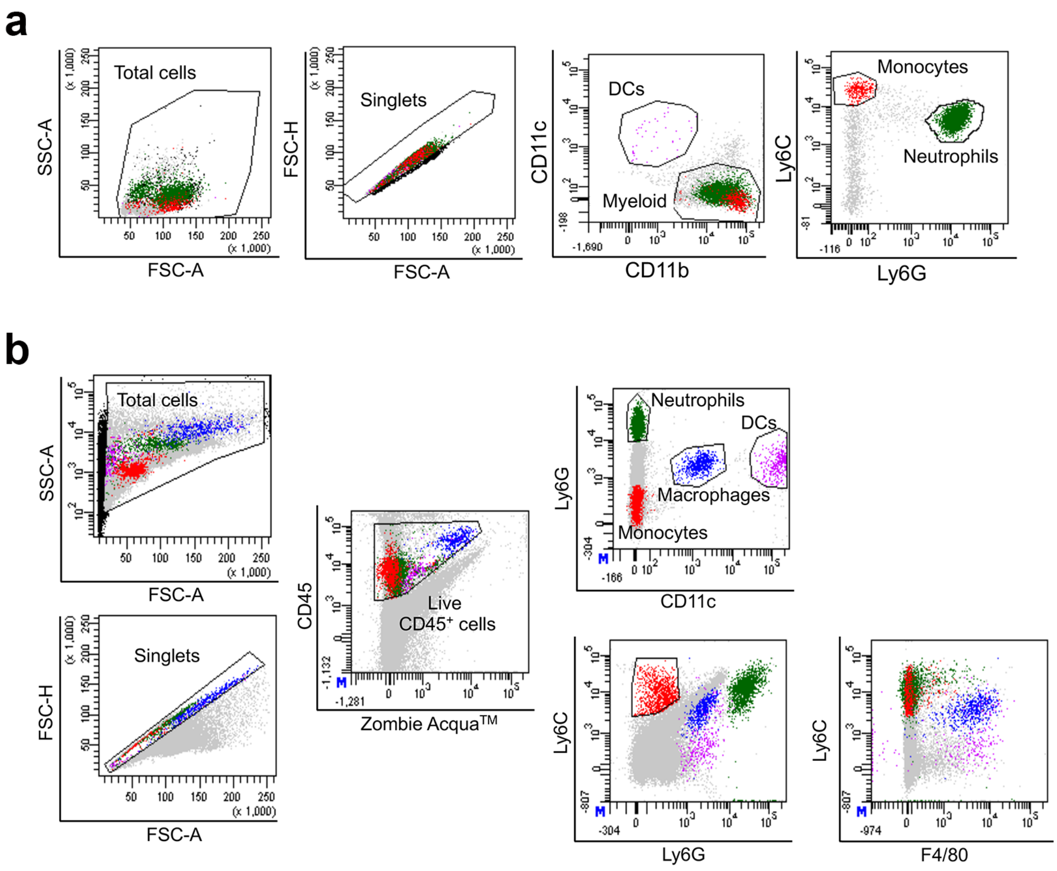

Supplementary Figure 13. **FACS gating strategy**. Identification of myeloid cell subsets by FACS in whole blood (**a**) and BALF (**b**) stained and analysed as described in Material and Methods section.
