# Supplementary material for: Serum amyloid P component is an essential element of resistance against Aspergillus fumigatus
Source: Nat Commun. 2021 Jun 18;12:3739. doi: 10.1038/s41467-021-24021-y (PMC8213769; doi:10.1038/s41467-021-24021-y)
Supplement: Supplementary file 3 — Reporting summary [file 41467_2021_24021_MOESM3_ESM.pdf]

nature research

Corresponding author(s): Alberto Mantovani

Last updated by author(s): 2021/04/20

Reporting Summary

Nature Research wishes to improve the reproducibility of the work that we publish. This form provides structure for consistency and transparency in reporting. For further information on Nature Research policies, see our Editorial Policies and the Editorial Policy Checklist.

Statistics

For all statistical analyses, confirm that the following items are present in the figure legend, table legend, main text, or Methods section.

n/a	Confirmed	
☐	☒	The exact sample size (*n*) for each experimental group/condition, given as a discrete number and unit of measurement
☐	☒	A statement on whether measurements were taken from distinct samples or whether the same sample was measured repeatedly
☐	☒	The statistical test(s) used AND whether they are one- or two-sided<br>*Only common tests should be described solely by name; describe more complex techniques in the Methods section.*
☐	☒	A description of all covariates tested
☐	☒	A description of any assumptions or corrections, such as tests of normality and adjustment for multiple comparisons
☐	☒	A full description of the statistical parameters including central tendency (e.g. means) or other basic estimates (e.g. regression coefficient) AND variation (e.g. standard deviation) or associated estimates of uncertainty (e.g. confidence intervals)
☐	☒	For null hypothesis testing, the test statistic (e.g. *F*, *t*, *r*) with confidence intervals, effect sizes, degrees of freedom and *P* value noted<br>*Give P values as exact values whenever suitable.*
☒	☐	For Bayesian analysis, information on the choice of priors and Markov chain Monte Carlo settings
☒	☐	For hierarchical and complex designs, identification of the appropriate level for tests and full reporting of outcomes
☒	☐	Estimates of effect sizes (e.g. Cohen's *d*, Pearson's *r*), indicating how they were calculated

Our web collection on statistics for biologists contains articles on many of the points above.

Software and code

Policy information about availability of computer code

Data collection	SNP genotyping was performed in genomic DNA isolated from whole blood of patients enrolled in the IFIGEN study using the QIAcube automated system (Qiagen) as described in the Method section. The levels of SAP were measured in clinical specimens from adult hematological patients enrolled in the FUNBIOMICS study as described in the Method section.<br><br>To collect microarray data deposited on GEO platform, the R packeage GEOquery (version 2.50.5) was used. Remaining data retrieved from public repositories were downloaded from related ftp (or in-house) servers without additional software.
Data analysis	The probability of IPA according to APCS genotypes was determined using the cumulative incidence method and compared using Gray's test. Cumulative incidences of infection were computed with the cmprsk package for R version 2.10.1. Multivariate analysis was performed using the subdistribution regression model of Fine and Gray with the cmprsk package for R version 2.10.1.<br>In silico analyses on publicly available and newly deposited data were performed with the following software:<br>Microarray data analyses:<br>R environment (version 3.5.2)<br>Biobase package (version 2.42.0)<br>limma package (version 3.38.3)<br>RNA-Seq data analyses<br>STAR alignment and quantification tool (version 2.6.1) with GENCODE annotation (version 33)<br>R environment (version 3.5.2)<br>DESeq2 package (version 1.22.2)<br>Plots were performed within R with<br>ggplot2 (version 3.3.2)<br>Raw RNA expression files were aligned and quantified with STAR (version 2.6.1) on the GRCh38 genome guided by GENCODE annotation

(version 33). Gene summarized counts were processed in R, genes whose expression was minor than 2 reads were removed while the remaining portion was “vs. normalized” with the R package DESeq268. Plots were rendered with the R library “ggplot2”.
Bands obtained in Western blot analysis were quantified by Fiji-ImageJ (NIH, Bethesda USA; version 2.1.0/1.53.c).
Confocal mages were analyzed with Leica Application Suite X software (LASX; version 3.5.5.19976).
Image deconvolution was performed by Huygens Professional software (Scientific Volume Imaging B. V.; version 19.10).
The statistical analysis of the whole paper was carried out using Prism version v6 or 7c.

For manuscripts utilizing custom algorithms or software that are central to the research but not yet described in published literature, software must be made available to editors and reviewers. We strongly encourage code deposition in a community repository (e.g. GitHub). See the Nature Research guidelines for submitting code & software for further information.

Data

Policy information about availability of data

All manuscripts must include a data availability statement. This statement should provide the following information, where applicable:

- Accession codes, unique identifiers, or web links for publicly available datasets
- A list of figures that have associated raw data
- A description of any restrictions on data availability

All data needed to evaluate the conclusions in the paper are present in the paper and/or in the Supplementary Information and Data Source File. Additional data related to this paper may be requested from the authors.
In silico analyses were based on publicly available software, custom scripts might be requested to the authors.
Microarray-based computational analyses were performed on publicly available datasets derived from the Gene Expression Omnibus (GEO) platform under the accession ID GSE42519, GSE11430, GSE3037, GSE2935, GSE25211, retrieved following these links:
https://www.ncbi.nlm.nih.gov/geo/query/acc.cgi?acc=GSE42519;
https://www.ncbi.nlm.nih.gov/geo/query/acc.cgi?acc=GSE11430;
https://www.ncbi.nlm.nih.gov/geo/query/acc.cgi?acc=GSE3037;
https://www.ncbi.nlm.nih.gov/geo/query/acc.cgi?acc=GSE2935;
https://www.ncbi.nlm.nih.gov/geo/query/acc.cgi?acc=GSE25211.
RNA-Seq data were derived from in-house performed sequencing experiments and are publicly available under the accession ID GSE160351 and GSE163533 at the links: https://www.ncbi.nlm.nih.gov/geo/query/acc.cgi?acc=GSE163531; https://www.ncbi.nlm.nih.gov/geo/query/acc.cgi?acc=GSE163533.

Field-specific reporting

Please select the one below that is the best fit for your research. If you are not sure, read the appropriate sections before making your selection.

[x] Life sciences   [ ] Behavioural & social sciences   [ ] Ecological, evolutionary & environmental sciences

For a reference copy of the document with all sections, see nature.com/documents/nr-reporting-summary-flat.pdf

Life sciences study design

All studies must disclose on these points even when the disclosure is negative.

Sample size	For in vitro studies, sample size was determined on the basis of previous experience [Refs: Garlanda, C. et al. Non-redundant role of the long pentraxin PTX3 in anti-fungal innate immune response. Nature 420, 182-186 (2002); Moalli, F. et al. Role of complement and Fcg receptors in the protective activity of the long pentraxin PTX3 against Aspergillus fumigatus. Blood 116, 5170-5180 (2010)].<br>For animal studies, sample size was defined on the basis of past experience on infection models [Refs: Garlanda, C. et al. Non-redundant role of the long pentraxin PTX3 in anti-fungal innate immune response. Nature 420, 182-186 (2002); Moalli, F. et al. Role of complement and Fcg receptors in the protective activity of the long pentraxin PTX3 against Aspergillus fumigatus. Blood 116, 5170-5180 (2010)], in order to detect differences of 20% or greater between the groups (10% significance level and 80% power).<br>For studies on IPA patients all available data/samples were used and no calculation was performed on sample-size. Sample-size (number of IPA patients) was considered sufficient based on previous studies [Ref.Cunha, C. et al. Genetic PTX3 deficiency and aspergillosis in stem-cell transplantation. The N. Engl. J. Med. 370, 421-432 (2014)].
Data exclusions	GraphPad Prism software was used to analyze the data. ROUT method (Q=1%) was applied to exclude outliers when suggested by GraphPad Prism. As indicated in Source Data File, a value in Figure 4C and 7D, two values in the controls in Figure 7C were excluded because they were identified as outliers.
Replication	All experiments were repeated as indicated in the Figure Legend.<br>Most experiments were replicated several times with reproducible results as indicated in Figure Legend/Statistics and reproducibility.
Randomization	Mice were randomized based on sex, age and weight. For the in vitro studies concerning cells or conidia, randomization was not relevant for those experiments because cells or conidia originated from same culture and divided in equal experimental groups.
Blinding	Allocation was blinded during data collection for in vivo experiments and in CFU counting. Genotyping of patient samples was also performed blindly.

Reporting for specific materials, systems and methods

We require information from authors about some types of materials, experimental systems and methods used in many studies. Here, indicate whether each material, system or method listed is relevant to your study. If you are not sure if a list item applies to your research, read the appropriate section before selecting a response.

Materials & experimental systems

n/a	Involved in the study
☐	☒ Antibodies
☒	☐ Eukaryotic cell lines
☒	☐ Palaeontology and archaeology
☐	☒ Animals and other organisms
☐	☒ Human research participants
☒	☐ Clinical data
☒	☐ Dual use research of concern

Methods

n/a	Involved in the study
☒	☐ ChIP-seq
☐	☒ Flow cytometry
☒	☐ MRI-based neuroimaging

Antibodies

Antibodies used

FACS:
- rat anti-CD45-PerCP , #30-F11, cat. #103130, lot. B218549, Biolegend; or anti-CD45-BV/650, #30-F11, cat. #563410, lot. 4329655, BD Biosciences;
- rat anti-Ly6G-PE-CF594, #1A8, cat. #562700, lot. 8215554, BD Biosciences; or anti-Ly6G- FITC, #1A8, cat. #11-9668-82, lot. 4335558, eBioscience-Invitrogen;
- rat anti-CD11b-BV421, #M1/70, RUO, cat. #562605, lot. B227048, BD Biosciences; or anti-CD11b-APC-Cy7, #M1/70, cat. #557657, lot. 9340080, BD Biosciences;
- rat anti-Ly6C-BV421, #AL-21, cat. #562727, lot. 6320819, BD Biosciences;
- hamster anti-CD11c-APC, #HL3, cat. #550261, lot. 5335829, BD Biosciences;
- rat anti-F4/80-PE/Cy7, #BM8, cat. #123110, lot. B237342, Biolegend;
- rat anti-murine SAP, #300103, cat. #MAB2558, lot. #VLM0407071, R&D Systems;
- goat anti-rat IgG AlexaFluor 647, #A21247, #lot. 903807, (ThermoFisher Scientific-Molecular Probes).

Western blot:
- goat polyclonal anti-C3, cat. #204869, lot. D00135263, Merck-Millipore;
- mouse anti-vinculin #hVIN-1, cat.#V9264, lot. 066M4757V, Sigma-Aldrich.
- mouse anti-human SAP, #910119, cat. # MAB1948, lot. CIVT0115061, Merck-Millipore;
- HRP-conjugated donkey anti-goat IgG, cat. #HAF-109, lot. XGD1117021, R&D Systems;
- HRP-conjugated sheep anti-mouse IgG, cat. #NA931V, lot. 168955895, GE Healthcare;
- HRP-conjugated goat anti-human IgG, #109-006-097, lot. 68379, Jackson ImmunoResearch.

Complement deposition:
- goat polyclonal anti-C3, cat. #204869, lot. #D00135263, Merck-Millipore;
- rat anti-mouse C1q, #7H8, cat. #HM1044, lot. #GR3183633-5, HyCult Biotech;
- rabbit anti-human C5b-C9 (MAC), cat. #A227, lot. #5b, Complement Technology, Inc.;
- goat IgG, cat. #026202, lot. #804535A, ThermoFisher;
- rat IgG2a, cat. #RTK2758, lot., B151064, Biolegend;
- rabbit IgG, cat. #ab27478, lot. # 684058, AbCam.

Blocking experiments:
- mouse anti-CD16 (FcgRIII), #3G8, cat. #302002, lot. #B254382, BioLegend;
- mouse anti-CD32 (FcgRII), #FUN-2, cat. #303202, lot. #244801, BioLegend; or anti-CD32 (FcgRII) #AT10, cat. #MABF925, lot. #2993682, Merck-Millipore;
- mouse anti-CD64 (FcgRI), #10.1, cat. #14-0649-82, lot. #4342644, eBioscience-ThermoFisher;
- mouse IgG1, #MG1-45, cat. #401402, lot. #B245259, BioLegend;
- mouse anti-M-ficolin, #036 051 1, cat. #sc-80486, lot. H2307, Santa Cruz Biotechnology;
- mouse anti-L-ficolin, #FCN219, cat. #sc-80484, lot. H2307, Santa Cruz Biotechnology;
- mouse IgG1, #P3.6.2.8.1, cat. #16-4714-82, ThermoFisher Scientific.

SAP depletion from human plasma:
- rabbit anti-human SAP, cat. #565191, lot. D00134885, Merck-Millipore (cross-linked on protein G-Sepharose beads).

Microscopy:
- rabbit anti-human SAP, cat. #565191, lot. D00134885, Merck-Millipore;
- rat monoclonal anti-C3 and activation fragments, #2/11, cat. #HM1065, lot. 23152M1017-A, Hycult Biotech (previously conjugated with Alexa Fluor647 through the Antibody Labeling kit (ThermoFisher Scientific-Molecular Probes);
- rat anti-Ly6G-BV421, #1A8, cat. #562737,BD Biosciences;

- rat IgG2A-BV421, cat. #562602,BD Biosciences;
- rat IgG2A-Alexa 647, cat. #557690,BD Biosciences;
- goat anti-rabbit IgG, Alexa Fluor 532, cat. #A11009, lot. 1833674, Invitrogen (ThermoFisher Scientific-Molecular Probes).

Validation

All antibodies used in the study are commercially available.

Following antibodies were used in FACS on mouse samples and tested in preliminary experiments with similar setting starting from manufacturer's instructions. Validation has been provided by the vendor:
rat anti-CD45-PerCP or rat anti-CD45-BV/650, #30-F11; rat anti-Ly6G-PE-CF594 or rat anti-Ly6G-FITC, #1A8; rat anti-CD11b-BV421 or rat anti-CD11b-APC, #M1/70; rat anti-Ly6C-BV421; hamster anti-CD11c-APC, #HL3; rat anti-F4/80-PE/Cy7, #BM8.
The staining protocol and FACS analysis are described in the Methods section and gating strategy shown in Supplementary Figure S13.
Omission of the rat anti-murine SAP, #300103 and detection with the goat anti-rat IgG AlexaFluor 647, #A21247was used as control.
The specificity of SAP binding on fungus is further established by competition assays with different proteins.

Following antibodies were used in microscopy on mouse lung samples as indicated by manufacturer's instructions and comparing with correspondent irrelevant IgG. Validation has been provided by the vendor:
rabbit polyclonal anti-SAP; Alexa Fluor647-conjugated rat monoclonal mAbs anti-C3, #2/11; BV421-labelled anti-Ly6G, #1A8.
Specificity was ascertained through the use of irrelevant control isotypes (BV421- and Alexa Fluor 647-labelled rat IgGs) and validated in lungs derived from SAP-deficient mice.

Following antibodies were used in Western blot analysis on lung lysates of mouse as indicated by manufacturer's instructions.
Validation has been provided by the vendor:
goat polyclonal anti-C3; mouse anti-vinculin #hVIN-1; mouse anti-human SAP, #910119.
Specificity is determined by the correct visible height of the bands obtained and the loading of positive controls.

Following antibodies were used in FACS analysis to evaluate complement deposition of human sera on conidia and used as indicated by manufacturer's instructions. Validation has been provided by the vendor:
goat polyclonal anti-C3, cat. #204869; rat anti-mouse C1q, #7H8; abbit anti-human C5b-C9 (MAC), cat. #A227. The specific differences in complement deposition obtained in the plasma of different mouse genotypes are supported by the use of sera depleted of complement molecules.

Following antibodies were used in blocking experiments in human neutrophils as reported in the literature (Moalli et al, 2010).
Validation has been provided by the vendor:
anti-CD16 (FcgRIII), #3G8; anti-CD32 (FcgRII), #FUN-2; anti-CD32 (FcgRII) #AT10; anti-CD64 (FcgRI), #10.1; irrelevant mouse IgG were used as control. Mouse anti-M-ficolin, #036 051 1 and mouse anti-L-ficolin were used with the correspondent irrelevant igG.

Following antibody was used for SAP depletion from human plasma. Validation has been provided by the vendor:
- rabbit anti-human SAP, cat. #565191; actual SAP depletion was ascertained after elution of proteins bound to resin.

Animals and other organisms

Policy information about studies involving animals; ARRIVE guidelines recommended for reporting animal research

Laboratory animals

Wild-type C57BL/6J, and Apcs-/-, C1q-/-, Mbl1/2-/- and C3-/- mice on a C57BL/6J genetic background, between 8 and 10 weeks of age, male of female were used. Wild-type C57BL6/NJ and Fb-/- mice on C57BL6/NJ on genetic background, between 8 and 10 weeks of age, male of female were used.
Wild-type mice on C57BL/6J and C57BL/6NJ genetic background were purchased from Charles River Laboratories (Calco, Como, Italy);
Apcs-/- mice were generated as described (Botto et al, Nat. Med. 1997) and providede by Prof. Marina Botto.
C1q-/- mice were kindly provided by Prof. John Lambris (University of Pennsylvania, USA).
C3-/-, Mbl1/2-/- and Fb-/- mice were purchased from Jackson Lab (Bar Harbor ME, US).

Wild animals

No wild animals were used in the study.

Field-collected samples

No field collected samples were used in the study.

Ethics oversight

Procedures involving animals and their care were conformed to protocols approved by the Clinical and Research Institute Humanitas (Rozzano, Milan, Italy) in compliance with national (4D.L. N.116, G.U., suppl. 40, 18-2-1992) and international law and policies (EEC Council Directive 2010/63/EU, OJ L 276/33, 22-09-2010; National Institutes of Health Guide for the Care and Use of Laboratory Animals, US National Research Council, 2011). The study was approved by the Italian Ministry of Health (approval n. 71/2012-B, issued on the 09/03/2012, 44/2015-PR issued 28/01/2015 and 742/2016-PR issued on the 26/07/2016).

Note that full information on the approval of the study protocol must also be provided in the manuscript.

Human research participants

Policy information about studies involving human research participants

Population characteristics	1, human peripheral neutrophils were isolated from peripheral blood of 30-50-year old male and female healthy donors, upon approval by Humanitas Research Hospital Ethical Committee.<br>2, clinical samples from hematological patients undergoing allogeneic hematopoietic stem-cell transplantation. A total of 483 hematological patients of European ancestry undergoing allogeneic HSCT at Instituto Português de Oncologia, Porto, and at Hospital de Santa Maria, Lisbon (Portugal), were enrolled in the IFIGEN study between 2009 and 2015. Clinical specimens from adult hematological patients enrolled in the FUNBIOMICS study at the Leuven University Hospitals, Leuven (Belgium). The cases of invasive aspergillosis were identified and classified as “probable” or “proven” according to the 2008 criteria from the European Organization for Research and Treatment of Cancer/Mycology Study Group (EORTC/MSG).<br>3, a cohort of patients admitted to Humanitas Clinical and Research Center (Rozzano, Milan, Italy) with a laboratory-confirmed diagnosis of COVID-19.
Recruitment	All patients transplanted in the period 2009-2015 in the involved centers were enrolled. Exclusion criteria included "possible" aspergillosis, infection with invasive moulds other than Aspergillus spp. or history of pre-transplant mould infection.
Ethics oversight	1, approval from Humanitas Research Hospital Ethical Committee.<br>2, Approval for the IFIGEN study was obtained from the Ethics Committee for Research in Life and Health Sciences (CEICVS) of the University of Minho, Portugal (no. 125/014), the Ethics Committee for Health of the Instituto Português de Oncologia - Porto, Portugal (no. 26/015), the Ethics Committee of the Lisbon Academic Medical Center, Portugal (no. 632/014), and the National Commission for the Protection of Data, Portugal (no. 1950/015). The FUNBIOMICS study was approved by CEICVS and the Ethics Committee of the University Hospitals of Leuven, Belgium (no.126/014).<br>3, study on COVID-19 patients was approved by the Humanitas Clinical and Research Center local Ethical Committee (authorization 233/20).

Note that full information on the approval of the study protocol must also be provided in the manuscript.

Flow Cytometry

Plots

Confirm that:

- [x] The axis labels state the marker and fluorochrome used (e.g. CD4-FITC).
- [x] The axis scales are clearly visible. Include numbers along axes only for bottom left plot of group (a 'group' is an analysis of identical markers).
- [x] All plots are contour plots with outliers or pseudocolor plots.
- [x] A numerical value for number of cells or percentage (with statistics) is provided.

Methodology

Sample preparation	1, as indicated in Material and Methods section, BALFs were performed with 1.5ml PBS, pH 7.4, containing protease inhibitors and 10mM EDTA with a 22-gauge venous catheter. BALFs were centrifuged, and, after erythrocyte lysis with ACK solution, cells were resuspended in PBS, pH 7.4, containing 10mM EDTA and 1% heat-inactivated fetal bovine serum and stained with as reported.<br>2, mouse whole blood was collected with heparin from cava vein and stained as reported in Material and Methods section.<br>3, human neutrophils were isolated from fresh whole blood of healthy volunteers through separation from erythrocytes by 3% dextran density gradient sedimentation followed by Ficoll-Paque PLUS and 62% Percoll centrifugation. Blood monocytes were obtained by Ficoll-Paque PLUS and 46% Percoll. Purity, determined by FACS analysis on forward scatter/side scatter parameters, was routinely >98%.
Instrument	1, 2, BD FACS LSRFortessaTM Flow Cytometer (BD Biosciences).<br>3, BD FACS CantoTM II Flow Cytometer (BD Biosciences)
Software	1, 2, BD FACSDiva™ Software v.6.2.<br>3, BD FACSDiva™ Software v.6.1.1.
Cell population abundance	1, 2, at least 10.000 events of CD45+/Ly6G+, CD45+/Ly6C+/F4/80+, CD45+/CD11c+, cells were considered as cut-off for the the FACS analysis of phagocytosis.<br>3, at least 100.000 cells were used for FACS analysis of phagocytosis.
Gating strategy	1, 2, gating strategy is reported in Data Source file at Worksheet Figure 6 and Figure 7.<br>3, purity of human neutrophils (defined as FSC-Ahigh/SSC-Ahigh) and monocytes (defined as FSC-Adim/SSC-Adim) was determined by FACS analysis on forward scatter/side scatter parameters and was routinely >98%.

- [x] Tick this box to confirm that a figure exemplifying the gating strategy is provided in the Supplementary Information.
